# Editorial: Childhood Adversity and Life-Course Consequences

**DOI:** 10.3389/fpsyg.2022.967180

**Published:** 2022-07-11

**Authors:** Naixue Cui, Cheryl Zlotnick, Yang Li, Nadya Golfenshtein

**Affiliations:** ^1^School of Nursing and Rehabilitation, Shandong University, Jinan, China; ^2^The Cheryl Spencer Department of Nursing, Faculty of Social Welfare & Health Sciences, University of Haifa, Haifa, Israel; ^3^School of Nursing, The University of Texas at Austin, Austin, TX, United States; ^4^School of Nursing, University of Pennsylvania, Philadelphia, PA, United States

**Keywords:** childhood adversity, stress, education, mental health, parenting

Childhood adversity is common in different sociocultural contexts and related to adverse outcomes throughout the life course (Caspi et al., 2017; Hughes et al., 2017, 2021; Bellis et al., 2019). Research in the past several decades has increased our understanding about the behavioral, psychological, and physical health outcomes, and the socio-economic burden related to childhood adversity. More precise and consistent measurements of childhood adversity, as well as the use of mixed-methods and longitudinal studies, and recently developed approaches such as epigenetics have further expanded the field. Nine original research articles were collected in the present Research Topic. All attempted to disentangle the effects of heterogeneous forms of childhood adversities, and examine the possible underlying mechanisms that result in negative outcomes.

Five of the nine research articles focused on relatively healthy children in school settings. They assessed heterogeneous forms of childhood adversity, including daily stress, psychological abuse and neglect, physical abuse, school bullying, and other environmental and personal encounters.

Xue et al. assessed the prevalence and risk factors of school bullying among a large sample of Chinese children and adolescents and found that 17.3% of the participants reported school bullying against their peers, and ~7.8% reported cyberbullying. Parental involvement and high self-control were protective against bullying, whereas experiencing interparental conflict and risk behaviors were associated with more bullying behavior.

Based on the process model of stress and coping, Wu et al.'s study examined the relationship between daily stress (i.e., bullying, examination failure, study load, financial problems in the family, family conflict, and chronic physical illness in a family member) and behavioral problems among primary school students. Their findings showed that high levels of daily stress were associated with more behavioral problems; however, this relationship was buffered by high-quality family functioning and good class environment.

Li et al. used longitudinal data from 271 primary school children and found that psychological abuse and neglect predicted low academic achievement 6 months later through low learning engagement. In addition, they reported that family socioeconomic status (SES) moderated the relationship between learning engagement and academic achievement, so that this relationship was stronger among children with low SES.

Similarly, Shen used data from 15-year longitudinal dataset of 9–12 years old children at the baseline in China and found that cumulative childhood adversity was associated with low educational attainment that was further linked to poor mental health in later adulthood. Interestingly, this study constructed cumulative adversity using a different set of adversities, including socioeconomic hardship, family disruption, children's physical issues (chronic disease, insufficient breakfast, or myopia), and academic setbacks. This study also examined the specific effects of different types of adversity on mental health across different life stages and assessed potential gender effects.

Cui et al. analyzed the potential role of P300, a proxy indicator of the allocation of neural resources and neurocognitive processing capability, in the relationship between physical abuse and externalizing behavior among a sample of school children. They reported that physical abuse by mothers was associated with more child externalizing behaviors through the mechanism of allocating more attentional resources to novel cues, which was indicated by enhanced P300 amplitude to novelty cues in an auditory oddball task.

Similarly, Moreno-Manso et al. reported significant cognitive impairment among 61 youths who were receiving protective services due to child maltreatment, poor family function, and others, and found that cognitive impairment was associated with more emotional and behavioral problems. The two studies together elucidate the possible neurocognitive mechanism underpinning the relationship between childhood adversity, and emotional and behavioral consequences.

The study by Mey et al. focused on male drug addicts under mandatory drug rehabilitation in Malaysia and revealed that more childhood maltreatment experiences were related to low treatment motivation. They also found that forgiveness and self-efficacy were possible psychological pathways through which childhood maltreatment mitigated treatment motivation.

Zhang et al. examined the joint and independent effects of different forms of childhood abuse (emotional, sexual, and physical) on different outcomes among pregnant women. Using data from 1,825 pregnant women, they reported that a high cumulative score of childhood abuse was associated with severe prospective and retrospective memory impairment. Among pregnant women exposed to only one form of child abuse, only women with emotional abuse experience demonstrated significant memory impairment, signifying the importance of assessing childhood abuse during prenatal care.

Finally, the COVID-19 pandemic has increased the risk of parenting stress. Parents with childhood adversity may be predisposed to vulnerability during this stressful period. Supporting this notion, Clemens et al. collected data from an online survey during the first lockdown due to the COVID-19 pandemic and found that parents with more adverse childhood experiences (ACEs) were more likely to exhibit harmful parenting behaviors.

In view of the findings of this Research Topic, it seems clear that regardless of the age-group, type of population, or exposure to a single or multiple type of adversities, people who were exposed to childhood adversity were at high risk of academic, emotional, behavioral, and cognitive problems. Although preventions and interventions have been emphasized to address the issues of adverse childhood experiences, the implementation and enforcement of these measures seem unsatisfying as a significant proportion of the participants involved in the included studies still suffered from such experiences. In this sense, it is still necessary to raise awareness of the harmful consequences of childhood adversity and apply prevention and intervention measures. In addition, future advances in differentiating positive, tolerable, and toxic stress, revealing the complex mechanism of how childhood adversity gets under the skin and produces life-long consequences, and therefore, designating and implementing preventive and therapeutic early interventions is warranted.

## Author Contributions

NC drafted the editorial. CZ, YL, and NG checked against results, edited, and refined the materials within this editorial. All authors contributed to the article and approved the submitted version.

## Funding

This research is part of the grant of the National Social Science Fund (18CSH061) and the Seed Fund for Candidates for Qilu Outstanding Scholars by Shandong University (2018WLJH46).

## Conflict of Interest

The authors declare that the research was conducted in the absence of any commercial or financial relationships that could be construed as a potential conflict of interest.

## Publisher's Note

All claims expressed in this article are solely those of the authors and do not necessarily represent those of their affiliated organizations, or those of the publisher, the editors and the reviewers. Any product that may be evaluated in this article, or claim that may be made by its manufacturer, is not guaranteed or endorsed by the publisher.
